# Comment on: intra-aortic balloon pump can be used after acute type A aortic dissection repair

**DOI:** 10.1186/s13019-026-04054-4

**Published:** 2026-04-17

**Authors:** Vitor Mendes, Anna Shonina, Mourad Malki, Mustafa Cikirikcioglu, Christoph Huber

**Affiliations:** Division of Cardiovascular Surgery, Department of Surgery, Geneva University Hospitals, Rue Gabrielle-Perret- Gentil 4, Geneva, 1205 Switzerland

## Dear Editor,

We read with great interest the article by Tanaka et al. reporting their institutional experience with mechanical circulatory support (MCS) in patients undergoing surgical repair of acute type A aortic dissection (ATAAD) [1]. This study addresses a long-standing and controversial question: whether the intra-aortic balloon pump (IABP), traditionally considered contraindicated in aortic dissection, can be used safely after ATAAD repair – and whether such feasibility translates into meaningful clinical benefit. The authors should be commended for addressing this challenging and underexplored field.

Current major guidelines for aortic disease (ACC/AHA 2022) do not provide specific recommendations regarding IABP use after ATAAD repair [2].

In clinical practice, however, IABP is traditionally considered contraindicated in aortic dissection because of the theoretical risk of propagation, rupture, or worsening aortic regurgitation, as reflected in surgical society resources (e.g., AATS TSRA Primer) and traditional device teaching materials [3]. Tanaka et al. describe 20 patients supported with IABP after acute type A aortic dissection repair, with no device-related aortic complications.

However, this study has important limitations: it is retrospective, single-center, spans more than 20 years, and includes a relatively small cohort (*n* = 31 requiring support, 20 IABP, 11 ECMO) [1]. Case selection, changes in surgical practice, and unmeasured confounders likely influenced outcomes. The absence of immediate IABP-related complications is reassuring, but late aortic events may have been underestimated, as follow-up imaging was available in only a minority of patients (8/20). Furthermore, case reports exist describing aortic dissection as a direct complication of IABP, including a recent example of acute descending thoracic dissection shortly after device insertion, requiring endovascular repair [4]. At the same time, these observations must be interpreted with caution and within the methodological constraints of the study. This challenges established dogma and suggests that, under selected conditions, IABP may be safer than previously assumed.

These points are duly acknowledged by the authors, who also note that ECMO patients were generally more severely ill, complicating direct comparison with the IABP group. Moreover, given the rapid evolution of surgical techniques, perioperative care, and the introduction of alternative devices such as Impella during the study period, extrapolation of these findings to contemporary practice should be made cautiously. Although the authors mention Impella as a possible alternative, no patient in their series was supported with this device. Future prospective multicenter registries directly comparing different modalities of MCS (IABP, Impella, ECMO) after ATAAD repair will provide more robust data to guide clinical practice.

Conversely, isolated reports describe successful use of IABP after ATAAD repair without evidence of propagation, supporting the notion that, under highly selected conditions, the device can occasionally be applied safely [5, 6].

The reported in-hospital mortality of 55% in the IABP group is high, underscoring that even if anatomically safe, the clinical benefit of the device remains uncertain. This highlights the need for careful patient selection, taking into account factors such as true lumen positioning, corrected aortic regurgitation, and preserved right ventricular function. Thus, while this study provides a signal of feasibility and anatomical safety, the impact of IABP on meaningful clinical outcomes remains uncertain.

Recent data suggest that standardization of surgical protocols for ATAAD reduces reoperations and late aortic dilation, indicating that the safety and efficacy of MCS such as IABP may depend not only on the device itself, but also on the quality of the surgical repair and the rigor of postoperative surveillance [7].

In conclusion, Tanaka et al. provide valuable data that may prompt a re-examination of the traditional contraindication of IABP after ATAAD repair. Prospective, multicenter investigations are warranted to better define patient selection, technical requirements, and outcomes. Until such evidence becomes available, we believe that IABP use in this context should be considered cautiously and on a case-by-case basis.

## Data Availability

No datasets were generated or analysed during the current study.
